# DOMAIN-SPECIFIC SUBJECTIVE COMPLAINTS AND OBJECTIVE COGNITIVE SCORES IN OLDER ADULT CANNABIS USE

**DOI:** 10.1093/geroni/igae098.2651

**Published:** 2024-12-31

**Authors:** Elizabeth Anquillare, Juliamaria Coromac-Medrano, Adrianna Neiderman, Rachel Thayer

**Affiliations:** University of Colorado Colorado Springs, Colorado Springs, Colorado, United States; University of Colorado Colorado Springs, Colorado Springs, Colorado, United States; University of Colorado Colorado Springs, Colorado Springs, Colorado, United States; University of Colorado Colorado Springs, Colorado Springs, Colorado, United States

## Abstract

Research on the cognitive effects of cannabis among older adults remains sparse and equivocal. Subjective cognitive complaints are predictive of future objective cognitive decline; however, few studies have explored this relationship in older adults with cannabis use (OACU) and domain-specific relationships have yet to be investigated. This study aimed to identify relationships between subjective and objective cognition across attention, memory, and executive functioning in a pilot sample of OACU. Participants included OACU between 60 and 79 years (N=17, Mage=68.29, SD=5.61), of which 94% were White and 59% were women. An average 15.94 years of education (SD=2.38) was reported. Participants completed online questionnaires regarding subjective cognition and participated virtually in a brief cognitive assessment battery. Items from the subjective cognition questionnaires were separated by domain and correlations were run between domain-specific subscales and standardized scores on the objective cognitive tests. Worse performance on a switching task was negatively associated with subjective attention (r=-.59, p<.05), memory (r=-.63, p<.05), and executive function (r=-.62, p<.05). Additionally, worse performance on an inhibition task was negatively associated with better subjective attention (r=-.50, p<.05), memory (r=-.51, p<.05), and executive function (r=-.52, p<.05). Similar negative correlations between measures of subjective cognition and objective memory and attention performance demonstrated a medium effect size without achieving statistical significance. Overall, subjective cognition was associated with executive functioning performance more than memory or attention performance. This pattern suggests OACU tend to describe their cognitive function broadly. Providers may wish to ask more nuanced questions to ensure comprehensive understanding of subjective cognitive functioning.

